# Factors associated with severity of road traffic injuries, Thika, Kenya

**DOI:** 10.4314/pamj.v8i1.71076

**Published:** 2011-03-10

**Authors:** Eric Osoro Mogaka, Zipporah Ng’ang’a, Joseph Oundo, Jared Omolo, Elizabeth Luman

**Affiliations:** 1 Jomo Kenyatta University of Agriculture and Technology, Field Epidemiology and Laboratory Training Program and Ministry of Public Health and Sanitation, Kenya; 2 Institute of Tropical Medicine and Infectious Diseases, Jomo Kenyatta University of Agriculture and Technology, Nairobi, Kenya; 3 Field Epidemiology and Laboratory Training Program and Centers for Disease Control and Prevention (CDC), Nairobi, Kenya; 4 Field Epidemiology and Laboratory Training Program and Ministry of Public Health and Sanitation, Nairobi, Kenya; 5 Centers for Disease Control and Prevention (CDC), Atlanta, Georgia, USA

**Keywords:** Road traffic accidents, injury, Injury severity Score, Kenya

## Abstract

**Background:** Road traffic injuries continue to exert a huge burden on the health care system in Kenya. Few studies on the severity of road traffic injuries have been conducted in Kenya. We carried out a cross-sectional study to determine factors associated with severity of road traffic injuries in a public hospital in Thika district, Kenya. **Methods:** Road crash victims attending the Thika district hospital, a 265-bed public hospital, emergency room were recruited consecutively between 10th August 2009 and 15th November 2009. Epidemiologic and clinical information was collected from medical charts and through interview with the victims or surrogates using a semi-structured questionnaire. Injuries were graded as severe or non-severe based on the Injury Severity Score (ISS). Independent factors associated with injury severity were assessed using multivariate logistic regression. **Results:** The mean age of participants was 32.4 years, three quarters were between 20-49 years-old and 73% (219) were male. Nineteen percent (56/300) of the victims had severe injury. Five percent (15) had head injury while 38% (115) had fractures. Vulnerable road users (pedestrians and two-wheel users) comprised 33% (99/300) of the victims. Vulnerable road users (OR=2.0, 95%CI=1.0-3.9), road crashes in rainy weather (OR=2.9, 95%CI=1.3-6.5) and night time crashes (OR=2.0, 95%CI=-1.1-3.9) were independent risk factors for sustaining severe injury. **Conclusion:** Severe injury was associated with vulnerable road users, rainy weather and night time crashes. Interventions and measures such as use of reflective jackets and helmets by two wheel users and enhanced road visibility could help reduce the severity of road traffic injuries.

## Background

Road traffic injuries are an important cause of morbidity and mortality worldwide, especially in low and middle-income countries and are currently ranked 9th globally among the leading causes of disease burden, in terms of disability adjusted life years (DALYs) lost [1,2].

The total annual costs of road crashes to low-income and middle-income countries are estimated to be about US$ 65 billion, which is more than the total annual amount received in development assistance [3].Road crashes in developing countries are more than double that of developed countries at 13.4/100,000 and 32.2/100,000 people in Europe and Africa respectively [1].

Kenya has one of the highest road fatality rates in Africa at 68 deaths per 10,000 registered vehicles and between 45-60% of admissions to surgical wards in public hospitals are as a result of road traffic injuries [4] Currently, the only nationally available road crash figures in Kenya are based on data collected by the police who attend to road traffic crashes or have details reported to them. However, some road crashes are not reported to the police; particularly crashes involving “vulnerable road users" such as pedestrians, pedal cyclists and motorcyclists, as well as victims who have mild injuries. In addition, few police officers have received medical training; thus, injury severity is classified into one of only three broad categories: slight, serious or fatal. 

Further, most published hospital-based surveys on road traffic injuries in Kenya have been conducted in the capital city of Nairobi as record reviews without detail on the severity of the injuries. These limitations have contributed to lack of awareness about the magnitude of the road traffic injuries [5,6].We carried out a cross-sectional survey to provide more accurate information on injury type and severity among road crash victims presenting to a peri-urban public hospital in Kenya, and to determine the factors associated with injury severity among these patients. 

## Methods

The study was carried out in the Accident & Emergency Department (A & E) of Thika District Hospital. Thika district, located 50 km north of Nairobi, is an industrial and agricultural area with an estimated population of 650,000 and is traversed by one of the busiest highways in the country [7].Thika District Hospital is a 265-bed regional referral hospital; it is the main public hospital in the district, and a majority of residents seeking medical care visit the facility. 

Road traffic crash victims attending the hospital within 24 hours of the road crash between 10th August 2009 and 15th November 2009 were approached for inclusion in the study. Patients presenting more than 24 hours after the road crash and those who refused to give consent were excluded. Road crash victims who did not seek medical treatment at the hospital, including those who died on-site or before arrival to the hospital, were also not included in this study. 

A minimum sample size of 289 was determined to detect differences in proportions between victims with severe and non severe injury. Assumptions made in determining the sample size included a prevalence of severe injury among the road crash victims of 25%, precision of 5% and confidence level of 95%. A road traffic crash was defined as one which took place on a road that involved at least one moving vehicle. A two wheeled vehicle was defined as a motorcycle or bicycle. Vulnerable road users were defined as pedestrians and those using two wheeled vehicles; non-vulnerable users were vehicle occupants, including those in cars, vans, buses or trucks. Head injury was defined as a Glasgow comma scale of below 12. Road crash victims were consecutively recruited and data were collected using pretested, standardized, interviewer administered semistructured questionnaires after the participants’ initial medical care. The interviewers were final year nursing students and the questionnaire was in English with a Swahili translation. 

For road crash victims brought to the hospital unconscious, informed consent was first obtained from the patient’s attendant and then from the patient when the condition allowed. For those under 18 years of age, consent was obtained from both the participant and their guardian. Data were recorded from the patient or from the patient’s attendant if the patient was unconscious or disoriented. Information about the circumstances surrounding the road crash was ascertained based on the perception of the victim. Alcohol use was assessed based on self report and breath odor, as assessed by the interviewer. 

Clinical information on injury type and severity was recorded from the medical charts. Additional details were also obtained from police and medical staff when available. Injury severity was assessed using the Injury Severity Score (ISS) [8,9].Each injury sustained was assigned an Abbreviated Injury Score and allocated to one of six body regions (head, face, chest, abdomen, extremities, and pelvis). Highest Abbreviated Injury Scores from the 3 most severely injured body regions were squared and summed to produce the ISS [8,10].Injury severity was categorized for this study as severe (ISS≥9) and non-severe (ISS < 9). Data were validated, cleaned, analyzed using Epi-info (version 3.5.1, CDC, Atlanta, GA, USA). Chisquare and Fishers exact tests (for cells 

## Results

A total of 300 road crash victims were recruited, consented and interviewed; 99% of those recruited agreed to participate. Approximately 19% (56) of the road crash victims sustained severe injury. Most road crash victims were male (73%), and approximately half had at least primary level education. The mean age was 32.4 years (range 3-75 years), with 75% (225) aged 20-49 years and 12% aged ≥50 years. 

Majority (68%) of the victims were vehicle occupants, while two-wheel vehicle users comprised 18% (54) and pedestrians 15% (45). Eighty percent (240) of the victims were involved in crashes on highways; 61% (255) reported that they were either angled or head-on collisions. Among the victims who were either drivers, pedestrians, motor cycle or bicycle riders, 21% (n=105) were suspected to have used alcohol. 

Evacuation of the injured to hospital was by taxi or other private vehicle (89%), police vehicles (6%) or ambulance (3%). Twenty percent (60) of road crash victims presenting to the hospital were admitted following their injuries, 3% (8) died at the A & E and 3% (8) of the road crash victims were referred to other hospitals either because of injury severity or patient preference. Most (69%) of the injuries sustained among study victims were to the head and neck region followed by the lower (64%) and upper extremities (23%). Among those who sustained head injury (n=15), 73% were vulnerable road users (Table 1).

The median ISS was four among vulnerable road users and one among non-vulnerable road users (p=0.001). Differences in proportion of sex, mean age, occupation, and education status between those with severe injury and those with non-severe injury were not statistically significant (Table 2). On bivariate analysis, vulnerable road users were at higher risk of sustaining severe injury compared to vehicle occupants (OR 1.8, p=0.03). Road crashes in rainy weather (OR 2.9, p=0.01) and at night-time (OR 2.4, p=0.004) were also risk factors for severe injury, while being in a crash while travelling in a van or bus at the time of the crash was protective (OR 0.5, p=0.02) (Table 3). In multivariate logistical regression, vulnerable road users, road crashes in rainy weather, and night time crashes remained independent risk factors for severe injury (Table 4). 

## Discussion

This study shows that when injured, vulnerable road users are at risk of sustaining more severe injury compared to vehicle occupants. These findings are consistent with other studies in Africa and Asia [4,11].This could be due to the reduced protection of a metal shell for vulnerable users compared to vehicle occupants and the free mixing of slow-moving road users with fast-moving vehicles. Road traffic crashes during rainy weather were found to be associated with severe injury.This finding is similar to that reported in case-control studies in Iran and Hong Kong [12,13].This could be due to decreased visibility and slippery roads during rainy weather which reduces ability to slow down and therefore increases the likelihood of a high speed road crash. Night-time road crashes were also found to be associated with injury severity. This finding was similar to a case-control study in Iran [12].This could reflect the reduced visibility, faster driving at night and influence of alcohol use which slows response time. 

Majority of those injured in the study were males which is consistent with findings from other studies in Kenya and in other low-income and middle-income countries [5].This could possibly be due to the greater exposure to traffic of the males compared to females as drivers or riders and as frequent travelers in motor vehicles for work-related activities. Three-quarters of the road crash victims in this study were aged 20-49 years. Similar age distribution of road crash victims was reported in other studies in Eldoret, Kampala and in a Kenyan country epidemiologic review [4,14,15].The involvement of this economically active and productive age group can result in significant economic loss at individual, family and societal levels. 

The most common region of the body injured among the victims was the head and neck followed by the lower extremity. This is consistent with findings in New Delhi where the most common pattern of injury was head (18.9%) followed by fractures of lower limb (17.8%) [16].On analysis by road user type, vulnerable road users accounted for two-thirds of head injuries which is higher than 55% reported in a study at a national referral hospital in Kenya [17].In this study, the proportion of road crash victims travelling in two-wheeled was higher than 6% reported in a retrospective hospital based study in Nairobi and 12% in a prospective hospital-based study in Eldoret [4,18].

Comparatively, motor cycle crashes accounted for 80% of the road traffic injuries in Thailand and more than 50% of the traffic deaths in Malaysia [19].In these two Asian countries two-wheelers represent more than 50% of all registered vehicles [19] compared to Kenya where motor cycles comprised 7.7% of all licensed vehicles in 2005 (Kenya government unpublished data). Further, with an increasing proportion of motor cycle users it is likely that be a higher proportion of severe injuries may be experienced. Alcohol use by pedestrians and drivers/riders was lower than 40% reported among drivers in a hospital survey in Eldoret, Kenya and 30% in a survey of motorcycle riders in Ife-Ife, Nigeria [20,21].However, it is likely that alcohol played a greater role than was found in this study and the differences could be due variability in methods of determining alcohol use. 

The prompt and safe evacuation of road crash victims to a health care facility is critical in management of injuries [22].The evacuation of crash victims from the crash scene in this study was mostly by private vehicle which is similar to findings in Uganda and Kenya [14,23].The finding in this study that only 3% of the road crash victims were evacuated by ambulance would be expected because there is no organized pre-hospital emergency medical service in Thika district. Although the hospital has ambulances they mainly serve to transport patients between hospitals and are rarely involved in evacuating the injured from road crash scenes. 

Our findings are subject to some limitations. Pre-hospital deaths and road crash victims who did not seek medical care at the facility were not included in the study. The study was therefore not able to assess risk of road traffic injury among various road users and only compared severity of injury among those who attended the hospital. Information on the nature and circumstances of the road traffic crash was based on the road crash victims’ perception. This bias was minimized by corroborating the patient’s account with that of other patients involved in the same road crash and attending the hospital or the police. 

## Conclusion

In this study, injuries sustained by pedestrians, motor cycle and bicycle users were more likely to be severe compared to vehicle occupants. Provision of protected lanes for these vulnerable road users may help protect them from being hit by automobiles. Severe injuries were also more likely to occur during rainy weather and at night. Road traffic injuries are preventable and measures such as use of reflective jackets and helmets by two wheel users and enhancing road visibility with street lights and reflective paint could help reduce the severity of road traffic injuries. 

## Competing interests

The authors declare no competing interest. 

## Authors’ contributions 

All the authors listed in this article made contributions during the design of the study, data collection and interpretation, provided critique for intellectual content and gave final approval of the version submitted. 

## Acknowledgments

We would like to acknowledge the management of Thika District hospital, The Ministries of Health, Kenya and Jomo Kenyatta University of Agriculture and Technology for facilitating the carrying out of the study. 

## Figures and Tables

**Table 1:** Injuries sustained by victims of road crashes by road user type, Thika, Kenya, 2009

Body region injured*	**Vulnerable Users (n=99) No. (%)**	**Vehicle occupants(n=201) No. (%)**
**Head and Neck**		
Superficial injury	60 (60.6)	130 (64.7)
Laceration	4 (4.0)	2 (1.0)
Fracture	5 (5.1)	2 (1.0)
Head injury†	11 (11.1)	4 (2)
**Thorax and Abdomen**		
Superficial Injury	7 (7.1)	2 (1.0)
Fracture	9 (9.1)	8 (4.0)
**Upper Extremity**		
Soft tissue injuries	13 (13.1)	32 (15.9)
Fracture	9 (9.1)	16 (8.0)
**Lower Extremity**		
Soft tissue injuries	48 (48.5)	65 (38.3)
Lacerations	4 (4.0)	3 (1.5)
Fracture	32 (32.3)	41 (20.4)

^*^Not mutually exclusive, ^†^GCS <12

**Table 2:** Socio-demographic characteristics of victims of road crashes with severe injury and non-severe injury, Thika, Kenya, 2009

**Variable**	**Severe Injury(N=56) No. (%)**	**Non-severe Injury(N=244) No. (%)**	**p-value**
**Sex**			
Female	16 (28.6)	66 (27)	
Male	40 (71.4)	178 (73)	0.49
Mean Age (SD)	32.5(12.4)	32.4(13.4)	0.94
**Age-group (years)**			
<10	3 (5.4)	10 (4.1)	
10-19	3 (5.4)	24 (9.8)	
20-29	16 (28.6)	77 (31.6)	0.67
30-39	19 (33.9)	74 (30.3)	
40-49	10 (17.9)	29 (11.9)	
>49	5 (8.9)	30 (12.3)	
**Occupation**			
Formal Employment	19 (33.9)	90 (36.9)	
Informal Employment	26 (46.4)	105(43)	0.97
Minor	8 (14.3)	37(15.2)	
Unemployed	3 (5.4)	12 (4.9)	
**Education**			
None	0 (0.0)	6 (2.5)	
Post-Secondary	10 (17.9)	35 (14.3)	0.34
Primary	31 (55.4)	116 (47.5)	
Secondary	15 (26.8)	87 (35.7)	

SD=Standard deviation

**Table 3:** Bivariate Analysis of factors associated with injury severity in victims of road crashes, Thika, Kenya, 2009

**Variable**	**Severe injury(n=56)**	**Non-severe Injury ">(n=244)**	OR*	**p-value**
	**No. (%)**	**No. (%)**	**(95% C.I.)**	
Vulnerable users	25 (44.6)	74 (30.3)	1.8 (1.0,3.4)	0.03
Helmet use	4 (7.1)	17 (6.9)	0.6 (0.2,2.3)	0.34
Weekend crash	38 (67.9)	135 (55.3)	1.7 (0.9,3.2)	0.12
Highway crash	49 (87.5)	191 (78.3)	1.9 (0.8,4.5)	0.17
Rainy weather	13 (23.2)	23 (9.4)	2.9 (1.4,6.2)	0.008
Night time crash	28 (50.0)	72 (29.5)	2.4 (1.3,4.3)	0.004
Use of van/bus	17 (30.4)	119 (48.8)	0.5 (0.2,0.9)	0.02
Alcohol use	5 (20.0)	17 (6.9)	0.9 (0.3,2.8)	0.57
Seat belt use	14 (25.0)	85 (34.8)	0.8 (0.4,1.8)	0.74

^*^Odds ratio

**Table 4:** Multivariate logistic regression of factors associated with injury severity among road crash victims, Thika, Kenya, 2009

**Variable**	aOR^*^(95% C.I.)	**p-value**
Vulnerable users	2.0 (1.0,3.9)	0.04
Weekend crash	1.6 (0.7,3.4)	0.25
Highway crash	1.7 (0.7.4.3)	0.21
Rainy weather	2.9 (1.3,6.5)	0.007
Night time crash	2.1 (1.1,3.9)	0.02
Use of van/bus	0.7 (0.3,1.5)	0.31
